# Nurse education and understanding related to domestic violence and abuse against women: An integrative review of the literature

**DOI:** 10.1002/nop2.133

**Published:** 2018-03-12

**Authors:** Kafi Fraih Alshammari, Julie McGarry, Gina Marie Awoko Higginbottom

**Affiliations:** ^1^ School of Health Sciences The University of Nottingham Nottingham UK; ^2^ Faculty of Nursing Community Health Nursing and Mental Health Department King Saud University Riyadh Saudi Arabia; ^3^ Chair of the Domestic Violence and Abuse Integrated Research Group Social Futures in Mental Health Centre of Excellence Institute of Mental Health Nottingham UK; ^4^ The Mary Seacole Professor of Ethnicity and Community Health School of Health Sciences The University of Nottingham Nottingham UK

**Keywords:** Domestic violence and abuse, Intimate partner violence (IPV), nurse education, women

## Abstract

**Aim:**

The aim of this study was to explore previous literature related to nurses understanding of Intimate partner violence (IPV) or domestic violence and abuse (DVA) against women and to identify the gaps in nursing education so as to use the findings as a baseline to inform potential intervention strategies, curriculum development and outline implications for future nursing practice.

**Design:**

An Integrative review of literature.

**Methods:**

Studies were extracted through a search of the electronic databases, such as Science direct, EBSCO host and PubMed, to identify relevant evidences published between January 2000–January 2017. “Joanna Briggs Institute (JBI) tool” was used to review primary research studies.

**Results:**

Seventeen empirical studies were analysed. Findings supported four themes including: educational and training experiences, identification of IPV/DVA, curriculum and communication skills of nurses. Continued efforts are further needed to highlight and address IPV/DVA in nursing education and training, to scale up nursing understanding to respond and identify IPV/DVA appropriately in a clinical environment.

**Why is this research or review needed?**

- IPV/DVA is a global health problem mainly perpetrated against women of all ages from every society.
- Most of the nurses lack adequate knowledge in reacting to and identifying IPV/DVA against women.
- How nurses should be prepared to deal with IPV/DVA against women during their educational tenure is yet to be investigated.

**What are the key findings?**

- Nurses play a crucial role in recognizing IPV/DVA against women and in providing them practical, emotional and psychological support.
- Collected evidences indicate that nurses during their education do not receive sufficient instructive training about IPV/DVA, in their advanced education curriculum, to permit them to deal with, recognize and identify IPV/DVA in their future specialized practice.
- Nurse usually lack confidence in responding to IPV/DVA mainly due to limited training and educational experience, fear of offending, lack of effective interventions and communication skills.

**How should the findings be used to influence policy/practice/research/education?**

- Nurses should consider routine screening for women suffering IPV/DVA as a standard of care.
- The study supports the need for undergraduate nurses to receive interactive learning opportunities engaging the victims and training on IPV/DVA against women at multi‐agency levels to raise awareness and identify suitable interventions.
- Future research is needed to influence the nurse education by integrating post‐ and pre‐registration courses and preparation programs in the nursing curriculum related to the issues of IPV/DVA against women.

## 1 INTRODUCTION

Intimate partner violence (IPV) or domestic violence and abuse (DVA) (UK Home Office, 2013) refers to the victimization of an individual by an intimate companion (Usta, Antoun, Ambuel, & Khawaja, 2012). According to the World Health Organization (WHO), “intimate partner violence and domestic violence” are used interchangeably, wherein, the latter term may also encompass other forms of family violence such as children and older people and is not confined to intimate partners (World Health Organization, 2012). IPV/DVA influences different domains of social life severely by deteriorating financial and social relationships with friends, families, children and victims. Females having experienced, emotional, sexual or physical violence may suffer a series of health related issues and often in silence. It is also acknowledged that IPV/DVA can also be experienced by men both in heterosexual and same‐sex relationships as well as can be perpetrated by women too. However, DVA affects women more than men (Dardis, Dixon, Edwards, & Turchik, 2015). IPV/DVA experienced by women is documented extensively as a significant “public health issue”, both due to the acute mortality or morbidity associated with it, thereof, in long term, influences the health of women (Lewis‐O'Connor & Chadwick, 2015). Nurses are often the first point of contact in healthcare services, who frequently encounter women suffering from IPV/DVA. However, in healthcare contexts, they may not be able to recognize or support women presenting with IPV/DVA (McGarry and Nairn, 2015). Nurses should essentially be equipped with the most contemporary knowledge and training to categorize concerns and manage different patients appropriately. Nurse's practice warrants psychological and physical demands everyday as they get engaged in several tasks. Therefore, other than experience, a nurse needs to have a comprehensive skill set so that health care for women does not suffer (Ghayath, Al‐Sagobi, Alansari, El‐Shazly, & Kamel, 2010). Given the potential impact of future nurses in reducing IPV/DVA against women, this area warrants further investigation. This study will help in influencing future research and nursing educational practice together with identifying significant gaps in care and well‐being of women. Thus, an integrative review of literature was undertaken to explore past evidence related to IPV/DVA against women and to identify the gaps in nursing education to use the findings as a baseline to inform potential intervention strategies, curriculum development in addition to outlining implications for future nursing practice.

### 1.1 Background

The fundamental aspects that influence nurse assessments of IPV/DVA are reported extensively across the healthcare literature. In spite of various endorsements from the professional bodies and mandates for assessment in emergency departments internationally, studies points towards the lack of IPV/DVA enquiry in majority of the outpatient settings (Clark, Lynette, & Mary, 2017). The nursing assessment is considered to be one of the most compelling elements of communication with patients regarding domestic violence (Reis et al., 2010). Previous anecdotal experiences by nurses in different healthcare settings demonstrates that victims of domestic violence often discuss their experience related to violence if inquired about it in a non‐judgmental, empathic and direct way. Previous literature also identified different factors about why intervention with reference to identification and screening with IPV/DVA victims are not performed reliably (Alvarez, Fedock, Grace, & Campbell, 2016). The primary barriers identified by nurses include language barriers, cultural differences, fear of repercussions of obligatory reporting laws, frustration is associated with the futility of the responses of healthcare systems, history of personal exposure to abuse, low confidence in inquiring questions, fear of offending victims and lack of privacy, resource knowledge available for victims, time constraints and lack of training. Perceived patient‐related barriers consist of: fear of police involvement, lack of disclosure, socioeconomic factors, accessing care because of perpetrator's prevention, shame and fear of retaliation and absence of follow‐up on referrals (Beynon, Gutmanis, Tutty, Wathen, & MacMillan, 2012).

Previous studies on barriers to intervene and screen with the IPV/DVA victims are abundant (Ahmad, Ali, Rehman, Talpur, & Dhingrra, 2016; Sprague, Madden and Simunovic, 2012). Educational needs are identified to be the most prominent preceding aspect (Crombie, Hooker, & Reisenhofer, 2017). Education that starts in schools of nursing continues during the course of the nurse career that helps to prepare nurses what is required for a sustained IPV/DVA practice. Education is the basic tool or facilitator needed to influence the integration of routine screening by nurses in practical settings. Protocols, routine screening queries included in the forms of assessments and chart prompts are some other facilitator that increases the screening process (Crombie et al., 2017).

IPV/DVA is a massive health problem globally. Domestic Violence cases are often reported and then, collected evidence is submitted for investigation. However, less attention is placed on the skills required by nurses to ensure that they are capable to properly screen women for the signs of abuse (Ramsay et al., 2012). Registered nurses have an imperative role to play in identification, responding, intervention and referral of women with current or past histories of IPV/DVA, however, more than one‐third of nurses have been found to have no formal education on IPV/DVA. Furthermore, research has found that there has been a shortage of content in nursing curriculum that is related to domestic abuse and violence against women. There is a dire need for educational intervention to ensure that nurses are provided appropriate training to equip them with adequate skills and knowledge in dealing with abused women. Since, limited or no medical curricula comprehensively covers DV‐related issues such as intervention strategies, medical consequences of DV and legal rights of females, therefore, this study will help to explore the gaps in nursing education with respect to IPV/DVA.

## 2 THE REVIEW/AIM

To explore previous literature related to nurses understanding with regard to IPV/DVA against women and to identify the gaps in nursing education to use the findings as a baseline to inform potential intervention strategies, curriculum development in addition to outlining implications for future nursing practice.

## 3 METHODS

### 3.1 Design

We conducted an integrative review using the theoretical framework espoused by Whittemore and Knafl (2005) to examine literature, including all methodological approaches and allow non‐experimental and experimental studies to inclusively comprehend the phenomenon being investigated. We used systematic and explicit process to reduce the risk of bias, improve the reliability of results together with usefully pooling data from both the empirical and theoretical evidences. This type of review is appropriate for our research because limited knowledge of nurses exists around this topic, therefore, the past theoretical or empirical literature will be reviewed that might lead to the conceptualization of a preliminary or initial topic (Torraco, 2005).

### 3.2 Search methods

Electronic databases including Science direct, EBSCO host, PubMed Ovid Medline database and Social Science Index were searched to extract relevant articles. The key word domestic violence/Intimate partner violence was combined with some other key terms such as “nurse”, “abuse” “knowledge”, “perception”, “understanding” “integrative review”, “education”, “curriculum” and “women” and the phrases used included “nurses and domestic violence”, “nurse education and domestic violence”, “nurses training and domestic violence” and “relationship between nurses and abused women”. Boolean connectors and truncation symbols “OR”, “AND” were used to merge terms for focusing and broadening the search.

### 3.3 Search outcomes

Findings of the papers written in English language published from the year January 2000–January 2017 were included, as majority of the studies related to nurse education on IPV/DVA were conducted over the last 17 years and is also considered as a golden period. This integrative review included both qualitative, quantitative and mixed method research studies. Articles that focused on nurses were included in the review whereas, studies involving midwives, other health professionals or doctors were excluded. The perception, understanding, attitude, knowledge and practice of nurse towards domestically abused women were included, however, studies on domestically abused men, children or workplace abuser were excluded. Furthermore, articles pertaining to the curriculum, skills, education, courses and training of nurses about IPV/DVA against women were also included (Table 1).

**Table 1:** Summary of integrative review databases, search terms, and inclusion criteria

Search terms	“Nurse”, “battering”, “abuse” “knowledge”, “perception”, “understanding” and “women” and the phrases used included “nurses and domestic violence”, “health care system and domestic violence”, “nurses training on domestic violence” and “relationship between nurses and abused women”.
Inclusion criteria	1. Published papers written in English language 2. Articles published in last seventeen years January 2000–January 2017 3. Articles focusing on nurses and nurse's student only (Excluding physicians and other health professionals) 4. Articles focusing on perception, understanding, attitude, knowledge, practice of nurses towards Domestic Violence against women only (Excluding men) 5. Articles focusing on curriculum, skills education, courses, training of nurses regarding DV against women
Databases	Science direct, EBSCO host, PubMed Ovid Medline database, and Social Science Index

### 3.4 Quality appraisal

To ensure external and internal validity of the selected studies, Joanna Briggs Institute (JBI) critical appraisal tool/Briggs institute checklist was used to examine the methodological quality of the qualitative and quantitative articles based on the level of evidence (Tables 2, 3, 4, 5). While for mixed method articles (Table 5), O'Cathain (2010) critical appraisal framework was used. These tools were used to characterize the strengths, limitation and impact of pertinent data with an intention of addressing the research aims/objectives and the quality of evidence being provided, mainly extracted in line with the specific objectives and review aims. We used this tool to lessen the risk of error/bias while pulling out data from each article individually.

**Table 2:** JBI critical appraisal checklist for qualitative research

	Checklist	Rigol‐Cuadra et al. (2015)	Duma (2007)	Inoue and Armitage (2006)	Woodtli (2001)	Guruge (2012)	Häggblom and Möller (2006)	Dedavid da Rocha et al. (2015)
1	Is there congruity between the stated philosophical perspective and the research methodology?	Unclear	Yes	Yes	Unclear	Yes	No	Yes
2	Is there congruity between the research methodology and the research question or objectives?	Yes	Yes	Yes	Yes	Yes	No	Yes
3	Is there congruity between the research methodology and the methods used to collect data?	Yes	No	Yes	Yes	Yes	Yes	Yes
4	Is there congruity between the research methodology and the representation and analysis of data?	Yes	Yes	Yes	Yes	Yes	Yes	Yes
5	Is there congruity between the research methodology and the interpretation of results?	Yes	Yes	Yes	Yes	Yes	Yes	Yes
6	Is there a statement locating the researcher culturally or theoretically?	No	Yes	Yes	Unclear	Unclear	Yes	Unclear
7	Is the influence of the researcher on the research, and vice‐ versa, addressed?	Unclear	Unclear	No	Unclear	Unclear	Yes	Yes
8	Are participants, and their voices, adequately represented?	Yes	Unclear	Yes	Yes	Yes	Yes	Yes
9	Is the research ethical according to current criteria or, for recent studies, and is there evidence of ethical approval by an appropriate body?	Yes	Yes	Yes	No	Yes	Yes	Yes
10	Do the conclusions drawn in the research report flow from the analysis, or interpretation, of the data?	Yes	Yes	No	Yes	Yes	Yes	Yes

Overall appraisal: Include yes.

**Table 3:** JBI critical appraisal checklist for analytical cross‐sectional studies

	Checklist	Glaister and Kesling (2002)	Tambağ and Turan (2015)	Bessette and Peterson (2002)	Cho et al. (2015)	Bryant and Spencer (2002)	Al‐Natour et al. (2014)	Davidov et al. (2012)
1	Were the criteria for inclusion in the sample clearly defined?	No	Yes	Yes	Yes	Yes	Yes	Yes
2	Were the study subjects and the setting described in detail?	Yes	Yes	Yes	Yes	Yes	Yes	Yes
3	Was the exposure measured in a valid and reliable way?	No	Yes	Yes	Yes	Yes	Yes	Yes
4	Were objective, standard criteria used for measurement of the condition?	Yes	Yes	Yes	Yes	Yes	Yes	Yes
5	Were confounding factors identified?	No	No	Unclear	Unclear	No	Unclear	Unclear
6	Were strategies to deal with confounding factors stated?	Yes	No	Unclear	Unclear	No	Unclear	Unclear
7	Were the outcomes measured in a valid and reliable way?	Yes	Yes	Yes	Yes	Yes	Yes	Yes
8	Was appropriate statistical analysis used?	No	Yes	NO	Yes	Yes	Yes	Yes

Overall appraisal: Include yes.

**Table 4:** JBI Critical Appraisal Checklist for Quasi‐Experimental Studies (Non‐randomized experimental studies) (Schoening et al., 2004)

No	Checklist	Schoening et al. (2004)
1	Is it clear in the study what is the “cause” and what is the “effect” (i.e., there is no confusion about which variable comes first)?	Yes
2	Were the participants included in any comparisons similar?	Yes
3	Were the participants included in any comparisons receiving similar treatment/care, other than the exposure or intervention of interest?	Yes
4	Was there a control group?	No
5	Were there multiple measurements of the outcome both pre‐ and post‐ intervention/exposure?	Yes
6	Was follow‐up complete, and if not, was follow‐up adequately reported and strategies to deal with loss to follow‐up employed?	Not applicable
7	Were the outcomes of participants included in any comparisons measured in the same way?	Yes
8	Were outcomes measured in a reliable way?	Yes
9	Was appropriate statistical analysis used?	Yes

Overall appraisal: Include yes.

Criteria for assessing the quality of Mixed Methods Research (O'Cathain, 2010).

**Table 5:** Critical appraisal checklist for assessing the quality of mixed methods research

Domain with quality	Items with domains	Davila, (2006)	Beccaria et al., (2013)
Domain 1: Planning Quality (Planning the study)	**Foundation**: Research questions and methods are based on sound examination of the relevant literature **Rationale**: Use of mixed methods approach is clearly justified and explained **Planning**: detail of intended design, data collection, analysis, and reporting is given **Feasibility**: Planned study can be completed with the available resources	√ √ √ √	√ √ √ √
Domain 2: Design quality (Conducting the study)	**Design transparency:** design is clearly articulated and related to known typologies where appropriate. **Design suitability**: design is suitable to answer research questions, and fits with other stated features i.e., reason for use of methods, paradigm **Design strength:** Selection of methods minimizes bias and enables broader/deeper study than single method **Design rigour**: Implementation of methods is congruent with study design	√ √ √ √	√ √ √ √
Domain 3: Data Quality (Conducting the study)	**Data transparency:** Detail is given of individual methods and their role in the study. **Data rigour/design fidelity**: Methods are rigourously implemented. Sampling adequacy: Selection approach and sample size are appropriate to method and context **Analytic adequacy**: Analysis is undertaken appropriately to the methods and questions **Analytic integration rigour**: Integration at the analysis stage, if conducted, is robust	√ √ √ √	√ √ √ √
Domain 4: Interpretive Rigour (Interpretation of data)	**Data transparency**: Detail is given of individual methods and their role in the study. **Data rigour/design fidelity**: Methods are rigourously implemented. Sampling adequacy: Selection approach and sample size are appropriate to method and context **Analytic adequacy:** Analysis is undertaken appropriately to the methods and questions **Analytic integration rigour**: Integration at the analysis stage, if conducted, is robust	√ √ √ √	√ √ √ √
Domain 5: Inference transferability (Interpretation of Data)	**Ecological transferability:** Inferences can be transferred to other contexts **Population transferability**: Inferences can be transferred to other populations **Temporal transferability**: Inferences are relevant to future contexts **Theoretical transferability**: Other data collection methods could be transferred.	√ √ √ √	√ √ √ √
Domain 6: Reporting Quality (Dissemination of Findings)	**Report availability**: successful completion of study within planned/allocated time and resource. **Reporting transparency**: key aspects of study are reported appropriately to the mixed methods design **Yield**: mixed methods design yields greater insight than single methods	√ √ √ √	√ √ √ √
Domain 7: Synthesizability (Real world application)	1. Qualitative element/study has qualitative objective or question 2. Qualitative element/study has appropriate design or method context for qualitative element/study is described 3. Sampling approach and participants in qualitative element/study are described 4. Approach to data collection and analysis in qualitative element/study is described 5. Researcher reflexivity in qualitative element/study is discussed Sequence generation or randomization in quantitative experimental element/study is appropriate 6. “Blinding” in quantitative experimental element/study is appropriate 7. Data sets are complete or largely complete in quantitative experimental element/study 8. Sampling and sample is appropriate to quantitative observational study/element 9. Choice of measurements in quantitative observational study/element is justified 10. Confounding variables are properly controlled in quantitative observational study/element 11. Mixed methods element/study is justified 12. Mixed methods element/study combines qualitative and quantitative data collection methods and/or analysis techniques 13. Mixed methods element/study integrates data or results from qualitative and quantitative elements	√ √ √ √ √ √ √ √ √ √	√ √ √ √ √ √ √ √ √ √
Domain 8: Utility	Findings are useful to “target audience” e.g., policy makers and consumers	√	√

Overall appraisal: Include yes.

### 3.5 Data abstraction

Hart (2000) demonstrated that analysis in any study is an aspect of systematically breaking down relevant data into different components and discussing how it relates with each other. Therefore, this study made use of thematic analysis as recommended by Aveyard (2010). It required to control the frequency of theme appearance or data type. To accomplish this, the investigator should be accustomed with the already collected data and must read the evidence being examined. Thematic analysis is considered to be the most suitable approach because it is an aspect of moving closer to the extracted data and mounting even deeper appreciation of the content (Fink, 2010). Thematic analysis was done based on the content similarity in addition to patterns observed in the chosen articles.

### 3.6 Data synthesis

**Figure 1:**
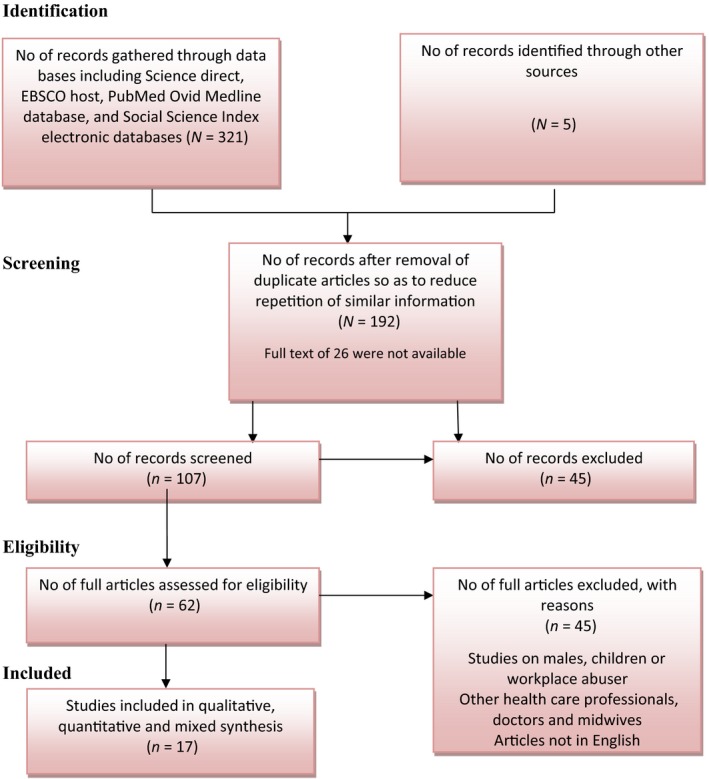
Schematic illustration of the selected studies

Hart (2000) indicated that data synthesis is an aspect of creating connections between the components being identified during analysis. It is referred to as an aspect where an investigator approaches literature as soon as it is assembled. The synthesis should be performed in a “step by step manner” to make the data more manageable. It is therefore imperative to have comprehensive subject knowledge and an aptitude to think in a broader context. It is undertaken by fetching together different studies as well as other pieces of information so as to discern new meanings. The final review yielded 17 studies as shown in Figure 1. The quantitative studies were dominated (*N *=* *8) (Table 6), only two studies used mixed method approach (*N *=* *2) (Table 7) and seven were qualitative studies (*N *=* *7) (Table 8). Moreover, themes were identified manually by the author (Table 9).

**Table 6:** Summary of Quantitative articles identified for data extraction and included in the integrative review

No.	Author/year	Purpose	Sample size & setting	Methodology	Findings/implications	Weaknesses and strengths
1	Glaister and Kesling (2002)	To determine the intervention and screening practices, perceived educational requirements and knowledge of legal requirements about interpersonal violence	251 Nurses/East Texas Area Health Education Center (AHEC)/Response rate 31%/ Random sample	Quantitative descriptive study/Survey	Clinical interaction, training and education with regards to violence varies among nurses. Study focused on the need of curriculum highlighting the need for training models to coordinate and share knowledge related to the prevention, intervention, identification and screening of interpersonal violence.	Better sample representation than non‐probability sample Lower response rate 31% Outcome measures were not addressed For respondents, competence was not defined
2	Tambağ and Turan (2015)	To examine the capability of nursing students, to identify signs of violence against females	259 nursing students/“Mustafa Kemal University School of Health Sciences in Hatay”, Turkey	Quantitative descriptive study/Survey	The capability of nursing students to recognize violence against females was insufficient. Development of courses on violence against women as well as its incorporation in the curricula of nurse students is needed in future	Sampling method not addressed Reliability of tool tested Ease of tool administration Economical methodology
3	Bessette and Peterson (2002)	To determine the ANP or adult nurse practitioner student's attitudes towards beliefs regarding DV along with determining how such beliefs and attitudes tends to impact the intervention and identification of women who experience such a violence.	34 ANP's/North Eastern United States/Convenience sample	Quantitative descriptive study/Survey	Results indicate that education of nurses is limited and has not prepared them to sufficiently care for battered females. Need for random sampling approach in diverse settings to provide generalized results Need to strengthen ANP curricula such as effectiveness and evaluation of programs that can implement clinical experience and course content about DV treatment, prevention and assessment.	Data acquired provided useful insights Generalizations is limited due to convenience sampling Possible sampling bias is possible since information was attained from students on a voluntary basis. Study lacks validity
4	Cho et al. (2015)	To categorize the beliefs and knowledge of nurses practicing in emergency rooms (ER) towards battering and violence	131 nurses with mean age 28.1 years old/Emergency center of 5 hospitals, located in Seoul and Gyeonggi‐do‐ South Korea/Convenience sampling	Quantitative descriptive method	Findings suggests ER nurse had limited knowledge about the interventions for DV, sexual assault tool kit and less educational experience about the concerns of abuse and violence	Study is limited by the location selected thus, affecting generalizability Violence situation of studies through a survey rather than observational data Larger sample size is warranted Reliability and validity of tool tested
5	Bryant and Spencer (2002)	To determine the practice behaviours of nurse practitioners about reporting, referring, identifying and inquiring of DV patients.	300 NPs, New York state/stratified random sample	Quantitative descriptive method/Survey	Significant difference was observed among family, adult, OB/GYN and women health NPs. OB/GYN and women health nurses were less aware of the screening questions about DV than other types of nurse practitioners. There exists a need to assess strategies that can encourage nurse practitioners to integrate universal DV screening behaviours in their practice.	A stratified random sample often provides greater precision Low response rate Study is limited by the location i.e., New York State thus, affecting generalizability; as it was unable to generalize all NP'S.
6	Schoening et al. (2004)	To examine the effects of IPV or intimate partner violence‐ educational program on nurse attitudes towards victims	52 inpatient nurses/An urban healthcare system/Convenience sample	A quasi‐experimental study/Pre and post‐test questionnaire	Intervention: one or three hours IPV education session PHNR scores of nurses increased if they possess prior IVP education and after attending a session of one hour. The scores of nurses increased after three hours of educational session only if they lacked prior IVP knowledge. Need for additional studies to address if alterations in the nurse attitudes translates into improved intervention, identification and screening for victims of IPV.	Relatively small sample Threat to internal validity‐lack of random assignment of nurses to either one or three hours of education session Lower post‐test return rate
7	Al‐Natour et al. (2014)	To regulate the IPV screening and barriers by nurses based in Jordan	125 nurses from all 3 Jordanian public hospitals and 10 out of 49 randomly selected public health clinics; Single Jordanian city/Stratified random sample”	Cross‐sectional design/Survey	The findings demonstrated a relatively lower rate of IPV screening among Jordanian nurses. No difference with regard to screening was observed between IPV victims when compared to non‐victimized nurses The barriers of IPV screening related to limited system support being the most essential clinical barrier.	Timely approach; Selection and measurement bias; Generalizations is limited due to sample collected from one city. Reliability and validity of tool was tested
8	Davidov et al. (2012)	To examine the nurse home visitors support for and attitudes towards obligatory IPV reporting between adults.	NFP nurses‐532 NFP sites; United States/Response rate of 49%	Quantitative, cross‐sectional research design/Survey	This study highlights the requirement to limit difference among physicians as well as establish reliable program practices mainly grounded in such principles complaint with state policies and existing researches.	Timely and inexpensive approach Low response rate Age of the client were not included

**Table 7:** Summary of mixed method articles identified for data extraction and included in the integrative review

NO	Author/Year	Purpose	Sample size & setting	Methodology	Findings/implications	Weaknesses and strengths
1	Davila (2006)	To recognize certain intimate partner violence skills and knowledge and shortfalls of nursing staff for the development of program. To assess the efficiency of training program on IPV skills and knowledge	53 program nurse's attendees, four licensed vocational nurses, 36 registered nurses, and one nurse practitioner/Response rate 48.8%	Mixed method Two phases “Phase 1: Qualitative approach for content development of in‐service program. Phase 2: 1‐group pretest–post‐test design to assess the program effectiveness”	“Findings supported the practice of an in‐service program as an operative means of increasing the IPV clinical skills of nurses. Further need to include violence related content within formal nursing curricula. The IPV training programs needs further evaluation mainly focusing on long term assessment to assess the sustained improvements of IPV skills and knowledge over time.”	Strength: use of mixed methods design Training program development founded on the adult learning theory Formal assessment of the program effectiveness Small convenience sample in Phase 2 Lower response rate
2	Beccaria et al. (2013)	To investigate the understanding and perceptions of the undergraduate students towards IPV	Survey: 62 respondents Focus groups: 27 nurse students	Mixed methods: Qualitative perspective; focus groups. Explorative quantitative; surveys	The results demonstrates that nurses are in a distinctive position to support, assist and identify women living with IPV, hence, huge understanding of nurse students attitudes and knowledge may help undergraduate programs to better guarantee nurse preparation for this role.	Stronger evidence for research conclusion (triangulation) Resource intensive Time consuming

**Table 8:** Summary of qualitative articles identified for data extraction and included in the integrative review

No	Author/year	Purpose & design	Sample size & setting	Methodology	Findings/implications	Weaknesses and strengths
1	Rigol‐Cuadra et al. (2015)	To assess views and beliefs of nursing students about exerted violence against married women	112 students, Between 19 and 35 years old. “Spanish university of Barcelona”, Tarragona University and Girona University/Convenience sample	Descriptive qualitative study Focus Groups, following Ecological Model	Nursing students interviewed possessed limited knowledge and training on the violence among couples. Study warrants a need for curriculum focusing on raising awareness about couple violence	Limitations not addressed Convenience sampling can be highly unrepresentative Potentially biased results due to group influence Results are subjective and not projectable
2	Duma (2007)	To assess how family violence was incorporates into the undergraduate nursing curriculums by making use of shelter as a setting for clinical learning	Six small groups of eight nursing students/Maryland USA/Purposive sampling	Descriptive qualitative case study/Document review, participant observation and interview	Usefulness of shelter for battered women was highlighted Conducive climate for learning is needed Highlighted the need for alternative clinical setting such as shelter for abused females	Time Constraints Scientific rigour of the study was affected Best suited to covers the context of the events occurred
3	Inoue and Armitage (2006)	To explore understanding of nurses’ regarding domestic violence issues by applying a grounded theory approach	41 emergency nurses/Australian and Japanese emergency departments	Qualitative/grounded theory approach/Face to face, unstructured interviews	The protocol and policy initiation and ongoing education provision enables a nurse to respond in a structured way when they encounter battered women.	Outcomes are grounded in data Theoretical sampling was used to guide data collection Validity addressed Sampling method description was addressed
4	Woodtli (2001)	To describe and explore the attitudes of nurses towards the perpetrators and survivors of domestic violence	Thirteen nurse participants; A large southwestern city; network sampling technique; Purposive sample	Qualitative interview study	Ecological Model for Health Promotion was supported in the as a valid holistic framework	Sample is confined to 1 city, thus limiting the generalizability of the findings Subjective and possibly selective retrospective recollections of respondents Standards for confirmability and authenticity of data was maintained
5	Guruge (2012)	To explore the role of nurses’ in caring for females who have experienced IPV	30 nurses; Sri Lanka; convenience sample	Qualitative interpretive descriptive design/Focus Groups	There is an urgent need for system of health care responding to the training needs and education of nurses in degree and diploma programs and need for continuous education	Trustworthiness and rigour was ensured Women perspectives about the care they had received was not addressed
6	Häggblom and Möller (2006)	“To explore the experiences of nurses supporting battered females, regarding violence against women and role of nurses as healthcare providers.	“10 female nurses; “government health organization in a local community” in Finland Purposive sample	Qualitative method based on grounded theory	The results confirmed that nurses encounters battered women even in small local communities	Generalizations is limited due to purposive sampling Larger sample size is warranted
7	Dedavid da Rocha et al. (2015)	To investigate the views of the nurse students from public university on including violence against women themes in their curriculum.	Federal de Santa Maria University; Southern Brazil, 18 graduate students of Nursing.	Descriptive exploratory qualitative research	Transversal theme of violence should be added in graduate nursing curriculum	His design has the ability to generate insights Sample is confined to 1 university, thus limiting the generalizability of the findings

**Table 9:** Some of the common themes extracted from the articles analysed as described in the findings are as follows:

Authors/years	Theme 1: Educational and Training Experience	Theme 2: Curriculum	Theme 3: Poor interviewing or communication skills	Theme 5: Identification of IPV (Intimate partner violence)
Rigol‐Cuadra et al. (2015)	X	X		
Duma (2007)		X		
Inoue and Armitage (2006)	X			X
Woodtli (2001)		X		
Guruge (2012)			X	X
Häggblom and Möller (2006)				X
Dedavid da Rocha et al. (2015)		X		
Glaister and Kesling (2002)	X			
Tambağ and Turan (2015)		X		
Bessette and Peterson (2002)	X			X
Cho et al. (2015)	X			
Bryant and Spencer (2002)				X
Schoening et al. (2004)	X			X
Al‐Natour et al. (2014)				X
Davidov et al. (2012)	X			X
Davila (2006)				X
Beccaria et al. (2013)			X	X

### 3.7 Ethics

Ethical approval was not required.

## 4 RESULTS

An initial search from the selected databases was performed to identify potentially relevant articles. This was followed by the abstract and title screening, after which full texts of potential studies were assessed and retrieved. A total of (*N *=* *321) and (*N *=* *5) studies were identified and examined for the construction of this paper. A total of 192 duplicate records were excluded to reduce repetition of similar information while full text of 26 were not available. After abstracts/title review, 107 full articles were reviewed for inclusion. 62 research papers were dated between 2000–2017. After thorough review of the full text; 45 articles were excluded from this investigation because it did not meet the inclusion criteria for instance, these were midwives studies or focused on children or males. The conduction of research strategy through MeSH terms, was performed that produced studies focused on midwives as well. These studies were excluded due to the lack of relevance to our proposed research question. We excluded midwives research studies with midwife participants because routine enquiry by midwives into domestic violence in UK is compulsory. The final review yielded 17 studies.

Evaluation of these articles found that the body of evidence on nurse knowledge on IPV/DVA was limited. The data were re‐categorized as well as recorded as essential to fit under more suitable headings as well as eventually, the categorized data were aggregated into four themes: educational and training experiences of nurses, identification of IPV/DVA, curriculum and communication skills of nurses.

### 4.1 Educational and training experiences of nurses

Participants in the majority of the studies demonstrated favorable attitudes towards the need of nurses for training and education on domestic violence during their undergraduate study (Bessette & Peterson, 2002; Cho, Cha, & Yoo, 2015; Glaister & Kesling, 2002; Guruge,^2012^; Inoue & Armitage, 2006; Schoening, Greenwood, McNichols, Heermann, & Agrawal, 2004). Nurses often need appropriate strategies as well as skills to respond to IPV/DVA to provide optimum care. Glaister and Kesling (2002) conducted a quantitative descriptive study with a random sample of 251 nurses indicating that DVA screening via routine inquiry is needed but should specifically be acquired by means of training and education along with guidelines development to aid the referral and identification in cases where domestic abuse is being disclosed. It is noteworthy for nurses to be aware that they are not required to be experts, however, they need to refer to some of the agencies. Multi‐agency information sharing is crucial to ensure IPV/DVA, specifically, cases of repeated victimization are recorded so that it is possible to put care measures in place as well as conduct risk assessment.

Schoening et al. (2004), undertook a quasi‐experimental study to inspect the effects of educational program on nurse attitudes towards DVA. The research took place in an urban healthcare system and included a convenience sample of 52 nurses. The study aimed to investigate if a training support program (one or three hours education session), that mainly targeted the nurses to escalate the identification of women who have experienced DVA. The convenience sampling method is considered to be the weakest form of sampling approach and noticeably is not capable to represent the general population.

The current knowledge of nurses were determined through scores identified from the “Public Health Nurses’ Response to Women Who Are Abused (PHNR)”, a standardized questionnaire that measures the reactions of nurses to IPV. Nurses expressed and supported the overall significance of education related to IPV/DVA for healthcare providers. A methodological strength of this study was the use of a pre‐ and post‐test design that can efficiently examine the effects of the educational intervention. Therefore, the nurses tend to lack domestic violence training and possessed limited knowledge, over and above were happy to get engaged with females experiencing IPV/DVA. Other studies such as Guruge (2012) in a qualitative interpretive descriptive study and Rigol‐Cuadra et al. (2015) in a descriptive qualitative study focused on the training needs, desirable for the ongoing support of women who are living with a violent companion. Guruge (2012) recruited a convenience sample of 30 nurses so as to determine the intervention and screening practices, perceived educational requirements and knowledge of legal requirements about interpersonal violence. The results emphasized on an urgent need for a healthcare system to respond to the training needs and continuous education of nurses to undertake degree other than diploma programs.

### 4.2 Identification of IPV/DVA

The identification of girls and women who are subjected to IPV/DVA is considered to be a criterion for adequate care and treatments along with specialized referral to services. IPV/DVA identification in healthcare hospitals can be improved if women are inquired about it, whereas, it is safe and effective only if followed by a suitable response (Al‐Natour, Gillespie, Felblinger, & Wang, 2014; Beccaria et al., 2013; Davidov, Nadorff, Jack, & Coben, 2012; Davila, 2006; Guruge, 2012; Schoening et al., 2004). This type of disclosure is relatively low to best predict the prevalence of IPV, while some studies indicate that in spite of training for universal screening, majority of the providers inquire about it selectively (Beccaria et al., 2013; Schoening et al., 2004).

Studies indicate that nurses needs to be knowledgeable and trained sufficiently in a variety of ways to respond as well as be conscious about the physical health indicators related to IPV/DVA, together with inquiring about violence when observed (Bessette & Peterson, 2002). Limited evidence reports about the universal screening policy. A qualitative focus group interviews conducted by Inoue and Armitage (2006) from 41 emergency nurses reported about the programs of screening showing limited evidence about the reductions in IPV/DVA, as well as health outcome improvements. Furthermore, in countries or settings where prevalence of IPV/DVA is high and scare referral choices are found, maybe a universal enquiry bring around limited advantage for women along with overwhelming health professionals (Bryant & Spencer, 2002).

Another study demonstrated that majority of women find queries regarding IPV/DVA tolerable (Schoening et al., 2004). Conversely, a study of 112 nurses, (between ages 19 and 35 years) showed that they are less enthusiastic to carry out routine analysis or screening of women who are to be interrogated. Disclosure of DVA is more expected if females are inquired in a non‐judgmental or empathetic way, in an environment or may be private where confidentiality is to be protected and an individual feels safe. Nurses can be offered training on how and when to inquire, as well as how to offer a first line response comprising of support, patient's experience validation, empathetic listening, in line with a female's desire (Rigol‐Cuadra et al., 2015). Hence, violence against women by their partners is an extremely stigmatized issue. Girls/women often have realistic fears for their protection if they unveil the DVA, due to which some situations needs to be met. These circumstances indicates that women should be inquired about the issue safely, when the abusive partner is not there. The nurses are trained recurrently in how to respond and inquire while, the protocols, referral systems and standard operating procedures are in place.

### 4.3 Curriculum

Attitudes of nurses regarding domestic violence, IPV/DVA related curriculum and learning experience of nurse have been a subject of the studies reviewed (Dedavid da Rocha, Celeste Landerdahl, Ferreira Cortes, Becker Vieira, & de Mello Padoin, 2015; Duma, 2007; Rigol‐Cuadra et al., 2015; Tambağ & Turan, 2015). The studies were mainly founded on the premise that an important approach for facilitating suitable care for women is via professional educational programs that prepares nurses who are skilled, knowledgeable and sensitive about the content linked to DVA. Moreover, it is urgently needed in any curriculum that prepares young nurses to practice. The studies principally focused on the possible impact of nurse feelings on the actions of nurses as well as questions the capability of a nurse to effectively intervene when some with a negative attitude forms the basis for the viewpoints of perpetrators and survivors (Rigol‐Cuadra et al., 2015).

Woodtli (2001) conducted a qualitative study using interviews to elicit data that described and explored the nurses attitudes towards the perpetrators and survivors of DVA. An ecological Model for Health Promotion was used in the study as a valid holistic framework. A network sampling technique was used to recruit a purposive sample of 13 nurse participants. Ecological model for health promotion was used as a curricula framework for DVA linked to practice and education. The results suggest that nursing education should prepare the nurses with adequate skills and knowledge to intervene at different levels such as public, community, institutional, interpersonal and personal policy. The methodological strength of this study was the use of purposive sampling that mainly targets highly homogeneous sample and was therefore considered best to answer the proposed research question. Other studies Duma (2007) used descriptive qualitative case study and Dedavid da Rocha et al. (2015) used a descriptive exploratory qualitative research design to validate the relative significance of a certain topic that can be judged by its significance with regard to the basic curricula of nurse qualifying courses. Another study conducted by Rigol‐Cuadra et al. (2015) and Duma (2007) provided several recommendations and suggestions regarding important curriculum content and necessary nursing skills proposed. However, these studies were qualitative and failed to draw on a rigourous scientific methodological genre such as ethnography and grounded theory etc.

### 4.4 Communication skills of Nurses

Beccaria et al. (2013) used a mixed method study design to survey 62 respondents and interview 27 nurse students. The study examined the understanding and perceptions of the undergraduate nurses towards DVA/IPV. Effective communication with women suffering IPV was also a statistically significant factor. The most required skill revealed by nurses was communication. The mixed method design used in the study included the benefits of both quantitative as well as qualitative approaches to research, often yielding better validity in the results. Conversely, Guruge (2012) conducted a qualitative interpretive descriptive design by recruiting a convenience sample of 30 nurses from Sri Lanka. This study highlighted the barriers to offer care such as threats to patient safety, language barriers, lack of communication and association between different stakeholders in health care.

Several limitations were found in this study that prevented the results from being generalizable. One included that the encounters with the women were not videotaped, which increased the likelihood of substantial non‐verbal communication. Duma (2007) supported such an insight that women were more likely to disclose their DVA experience only, if staff used open ended queries and introduced the topic for probing further violence and by inquiring about the follow‐up queries. This study also highlighted the need for an alternative clinical setting such as shelter for females suffering DVA.

## 5 DISCUSSION

The findings from the studies reviewed in this integrative literature review, evidenced nurses understanding with regard to domestic violence against women and identified the gaps in nursing education. These new insights will enable the findings to be used as a baseline to inform suitable intervention strategies and curriculum development (Beccaria et al., 2013; Bessette & Peterson, 2002; Cho et al., 2015; Davila, 2006; Dedavid da Rocha et al., 2015; Häggblom & Möller, 2006). Four themes were demonstrated in the findings such as educational and training experiences of nurses, identification of IPV/DVA, educational curriculum and communication skills of the nurses. To date, no integrative review exists highlighting the most common themes emerging from this review, comprising of gaps in the knowledge of nurses and education related to DVA/IPV, perceived system support and lack of effectual interventions. It is essential for nurses to share similar prejudices and cultural norms with women suffering from DVA/IPV that may have an impact on their professional attitudes (Guruge, 2012). Furthermore, a nurses may believe that DVA/IPV mainly against women is not only a health issue but a private family matter. Moreover, the allocated resources to this field are not sufficient, due to which few nurses may feel desperate, leading them to be reluctant professionally. Nevertheless, nurses often owns a commitment and opportunity to manage and identify the DVA/IPV (Al‐Natour et al., 2014; Glaister & Kesling, 2002).

The knowledge of DVA/IPV and its definition was considerably better among those screened than the people not being screened. The results of our study endorsed a requirement to improve nursing staff knowledge mainly about better communication and all other aspects of DVA such as social, legislative issues, referrals and interventions pertaining to DVA (Beccaria et al., 2013; Guruge, 2012). Nevertheless, the methods and duration of training and increased awareness often regards to the need for meticulous decisions and selections. Another notable finding from our review suggested positive professional trainings influencing the reported screening practices of DVA/IPV (Bessette & Peterson, 2002; Inoue & Armitage, 2006). Other studies demonstrated that professional training tends to have a positive on the DVA/IPV screening practices (Papadakaki, Petridou, Kogevinas, & Lionis, 2013). Nonetheless, studies directly examined the training effects that have developed conflicting consequences based on the length of follow‐up and training types. Similarly, results from international studies suggested that the professional training has a potential to enhance the skills, comfort and knowledge for effective intervention and inquiry (Krasnoff & Moscati, 2002). Thus, without sufficient structural modification, institutional policies and in‐service education provided regularly along with training is not likely to be adequate to alter the nursing practice. Moreover, controlled studies are required further to study the intervention effectiveness so as to improve the behaviour of nurses regarding the domestic violence.

Studies have also demonstrated that women might not disclose violence to nurses unless direct enquiry has been raised from nurses. The review findings primarily focused on the need to encourage women to create and disclose opportunities where they feel safer to talk regarding the experiences. Such results are also being echoed in other studies, with women undergoing a huge lengths in making efforts to avoid DVA/IPV disclosure (Beynon et al., 2012; Montalvo‐Liendo, 2009). Considerably, women make attempts to hide such violence due to the feelings of shame and being stigmatized (Spangaro, Poulos, & Zwi, 2011). The government of UK have responded to the concern of DVA in healthcare settings with a Resource Manual for Health Care Professionals (National Assembly for Wales, 2001) and Domestic Violence Training Manual publication (Department of Health (DH) and Home Office, 2006). Both of these publications perceives to identify a primary role for nurses as well as recommends mandatory training with a designated lead and continuous professional education.

Results from our review can be used to notify the implementation as well as development of “nursing curriculum” at undergraduate level on DVA (Dedavid da Rocha et al., 2015; Rigol‐Cuadra et al., 2015). There exists a necessity to offer opportunities for learning that imposes a challenge for pre‐existing assumptions as well as beliefs to guarantee that graduates are unable to embrace narrow definition of DVA. At a wider level, given the evidence, it is important that undergraduate nursing programs both at international in addition to national context addresses the significance of nurse role around DVA/IPV as well as continues to focus on addressing the stereotypical conceptions about DVA (Cho et al., 2015; Schoening et al., 2004). This review also provides insights into undergraduate nursing opinions and attitudes regarding DVA. The findings highlights concern areas that are often comparable to the problems identified in the broad literature. These findings also has implications for nursing education ensuring that graduates are well equipped to suitably respond to and identify DVA/IPV in the clinical settings. Our findings also resonate with the WHO “Clinical and Policy Guidelines (2013) for Health Sector Response to Violence against Women” that mainly focused on the need for training at “pre‐qualification level.” These guidelines aims to raise awareness about the DVA/IPV against women among policy makers together with health professionals so that they can adequately comprehend the necessity for suitable responses in a healthcare environment. A standard offered in a certain guideline can be incorporated into the education of nurse professionals.

### 5.1 Strengths and limitations

The integrative review was strengthened using a specific framework for guidance. Our review was enhanced by implementation of a widely accepted critical appraisal tool “JBI” to assess the quality of each study and huge number of international studies counted in our analysis. This review has numerous limitations which may lead to limit the generalizability of the findings. For example, grey literature was not searched. The exclusion of studies in languages other than English language also served as a limitation that resulted in exclusion of some substantial and important evidence. Although this review was systematic in our review steps and processes, but was not a systematic review. Adoption of a systematic review approach would have enhanced the scientific rigour of review, however, limited financial and personnel resources prevented us from adopting this approach.

## 6 CONCLUSION

Intimate partner violence (IPV) or domestic violence and abuse (DVA) often has a huge psychological or physical impact on the lives of women. Major success for implementation of a DVA enquiry is based on the developmental needs and training of the nurses involved. Nurses tend to play a statistically significant role in recognizing individuals who are DVA victims, boosting the developments of safety plan as well as expediting access to support on top of assistance. Courses at undergraduate level in a nursing university is an ultimate opportunity to bring changes in the attitudes concerning DVA, also equip nurses with an inclusive understanding of IPV/DVA. This review offers insights into the opinions of nurses on IPV/DVA as well as identifies a need for constant consideration to address persistent educational needs to identify DVA against women. This review draws attention to the need for future research to influence the nurse education on shaping suitable professional attitude towards IPV/DVA as well as influence clinical practices.

### 6.1 Implications for practice, education and research

The findings of this review may be of significance to nursing practice, as nursing staff caring for women suffering from DVA can be facilitated in developing a positive attitude towards the use of screening questions, better communication methods and advanced training during the course of their study. Nurses often have a duty of care towards their patients/clients, yet IPV/DVA is thought to be largely unreported or unasked, with many of the women going home with the DVA perpetrator. It is important to consider that nurses are professionals who are often involved with the care of patients, which may create a safe environment for a disclosure to happen. Hence, there lies a need for mandatory collaboration between secondary and primary health care along with trainings, liaising with some local agencies, to provide support to women who experience DVA. It is essential to include post‐ and pre‐registration courses in nursing curriculum. Cooperative networking are required for establishing the multi‐agency guidelines and trainings, all of which tends to influence the DVA identification. Referral channels and information sharing need to be established in secondary and primary sectors for providing assistance in this process. Training at multi‐agency levels are needed to: raise awareness, identify, estimate risk levels, document as well as identify suitable intervention levels.  

## CONFLICT OF INTEREST

No conflict of interest has been declared by the author(s).

## AUTHOR CONTRIBUTIONS

“All authors have agreed on the final version and meet at least one of the following criteria [recommended by the ICMJE (http://www.icmje.org/recommendations/)]:

- Substantial contributions to conception and design, acquisition of data or analysis and interpretation of data;
- Drafting the article or revising it critically for important intellectual content.”
